# Primary Anorectal Melanoma: A Case Report

**DOI:** 10.31729/jnma.8150

**Published:** 2023-05-31

**Authors:** Sajjad Ahmed Khan, Adarsha Neupane, Samit Kumar Gautam, Sulav Sapkota

**Affiliations:** 1 Birat Medical College Teaching Hospital, Biratnagar, Morang, Nepal; 2 Department of Medical Oncology, Birat Medical College Teaching Hospital, Biratnagar, Morang, Nepal

**Keywords:** *abdominoperineal resection*, *adjuvant chemotherapy*, *case reports*, *melanoma*

## Abstract

Primary anorectal melanoma is an extremely rare and aggressive mucosal melanocytic malignancy of the anorectal region. Because of the rarity of the tumor and vague clinical presentations, diagnosis at an early stage is a challenge for clinicians. In our context, where the hemorrhoid is a diagnosis of cultural familiarity for any sort of rectal pathology, these patients often present to us at a very advanced stage of the disease. Here, we present a case of a 55-year-old male patient with stage 2 anorectal melanoma who is on adjuvant chemotherapy following abdominoperineal resection with a permanent colostomy. Five cycles of dacarbazine and carboplatin have been given and the patient is doing well with the treatment. Abdominoperineal resection with excision of the tumor remains the mainstay of treatment; however, poor patient compliance with permanent colostomy is a major limiting factor of abdominoperineal resection. Even with the best interventions and care, the survival rate is not very good.

## INTRODUCTION

Melanoma is a tumor produced by the malignant transformation of melanocytes, which originates from neural crest cells.^1^ Anorectal melanoma is a rare entity accounting for 0.4% to 1.6% of total malignant melanomas.^2^ Being a highly malignant and aggressive tumor, the five-year survivability rate ranges from 10% to 20%.^3^ There is insufficient evidence on the optimal medical management of the disease and treatment typically involves a multimodal approach including surgical resection, chemotherapy, targeted therapy, and/or immunotherapy.^4^ In our reported case, we went for APR and the patient is on adjuvant chemotherapeutic agents.

## CASE REPORT

A 60-year-old male presented to us with chief complaints of difficulty in defecation and per rectal bleeding for three months. Difficulty in defecation was acute in onset, progressive, and associated with tenesmus of about 20 episodes per day with no history of any protruding mass out of the anus. Bleeding per rectum was fresh, scanty in amount, without passage of blood clots. Pain aggravated on sitting and relieved while lying down. There was also associated weight loss with no history of fever, abdominal pain, chest pain, cough, shortness of breath, hemoptysis, burning micturition, hematemesis, abnormal bodily movements, blackouts, and yellowish discoloration of the skin and mucous membrane.

The patient is a known case of hypertension diagnosed three years back and is controlled under medication. The patient smokes 20 sticks per day for the past 46 years which corresponds to a pack year of 46, but has left smoking since last year. Patient further gave a history of intermittent consumption of arrack for the past forty years. No history of diabetes mellitus, tuberculosis, chronic obstructive pulmonary disease (COPD), thyroid disorder, and any other chronic illness. No history of any surgical procedures in the past.

Suspecting it to be a case of hemorrhoids, the patient visited a local health center where he was given some topical medications which he continued for one month. Due to the persistence of symptoms despite one month of medication at the local health center, he visited us.

The patient was of average build, conscious, and oriented to time, place, and person. There was the presence of pallor. icterus, clubbing, cyanosis, and edema were absent and hydration status was normal. There was no palpable lymph node. Vitals were within normal limits.

On per abdominal examination, the abdomen was smooth, non-tender, and tympanic on percussion with normal bowel sounds heard on auscultation. Per rectal examination revealed a 4x3 cm ulcerated mass around the anal verge with normal prostrate felt on digital rectal examination (DRE). Cardiovascular and respiratory findings were normal.

**Figure 1:**
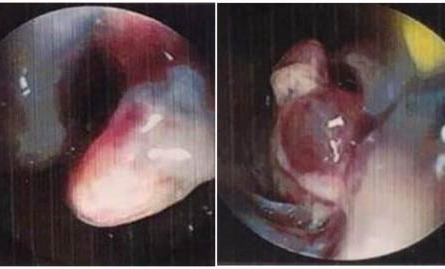
Colonoscopic view showing polypoidal, blackened growth.

Subsequently, a colonoscopy was performed which revealed polypoidal, blackened growth (Figure 1).

**Figure 2:**
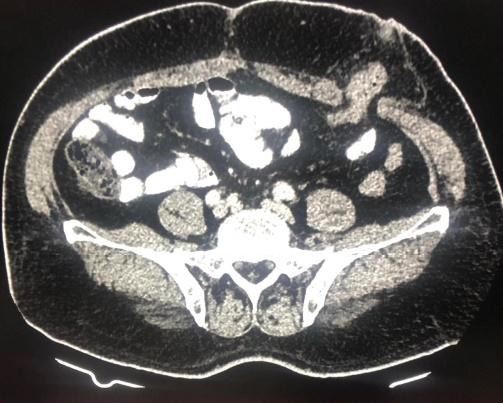
CT scan of abdomen showing end colostomy following APR.

On further radiological investigations, findings were suggestive of malignancy and hence abdominoperineal resection (APR) was planned (Figure 2).

**Figure 3:**
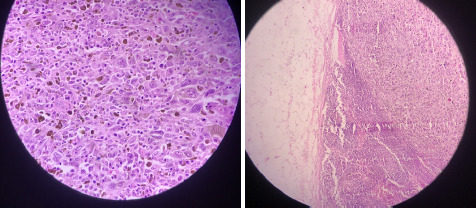
Histopathological evidence of malignant changes

End colostomy was done following APR and the patient is currently on adjuvant chemotherapy. Excisional biopsy was sent for histopathological evaluation which revealed anorectal melanoma stage 2 (Figure 3).

**Figure 4:**
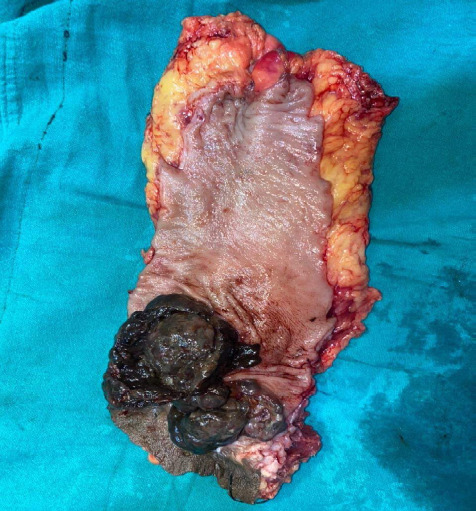
Cut surface showing solid greyish proliferative mass measuring 8 cm in diameter with edematous mucosa.

Following APR, the gross specimen on cut surface showed solid greyish proliferative mass from which exicisional biopsy was sent (Figure 4).

He has completed six cycles of dacarbazine and carboplatin and is doing well with it. At present, stoma care has become a matter of concern for the patient, in terms of hygiene and compliance. Similarly, mild dizziness and body aches are a few tolerable side effects seen after taking chemotherapy. Otherwise, the patient is doing well with the treatment and is able to give continuity to his daily routine activities.

## DISCUSSION

Our understanding of the pathophysiology of malignant melanoma is limited and obscure which might be a major limiting factor for the development of targeted therapies against the disease.^5^ In our setting, the inadequacy of diagnostic and therapeutic modalities, lack of accessibility to health centers, poor patient compliance, ignorance of any sort of rectal lesion as hemorrhoids and subsequent delay in seeking clinical care are a few inevitable factors that make the management approach further challenging.

In these groups of patients, operative management has been considered an effective approach, however, controversy still exists regarding the benefit of radiation therapy and chemotherapy.^6^ In our case, we did APR followed by loop ileostomy. After surgery, the patient is being kept on dacarbazine and carboplatin, as a part of adjuvant therapy. The exact mechanism of dacarbazine is not known but is thought to exert a cytotoxic effect by acting as an alkylating agent.^7^ Similarly, carboplatin also acts as an alkylating agent causing cross-linking between and within deoxyribonucleic acid (DNA) strands, leading to inhibition of DNA, riboxynucleic acid (RNA) and protein synthesis and the triggering of programmed cell death, mostly in rapidly dividing cells.^8^

It has been almost a year and the patient is currently in good health status, able to carry out his day-to-day activities without any significant toxic effects of these chemotherapeutic agents. A similar case was reported in China in which APR was done and no other adjuvant therapy was added, and the patient is doing well after 24 months of surgery.^9^ In our case, together with APR, we added adjuvant chemotherapy in the form of dacarbazine and carboplatin to prevent the recurrence of the disease. Being a very rare entity, little attention has been given to anorectal melanoma and management of the tumor is often a dilemma in the clinics. As per the recent evidence, abdominoperineal resection followed by post-operative chemotherapeutic agents is the mainstay of treatment, however, survivability is not very good, and life-long colostomy following APR further adds to the problem. In our setting, the scenario is even worse, lack of public awareness about the disease processes, ignorance about the clinical features at an early stage, presenting at a very advance stage and poor compliance to treatment and follow-up are a few other inevitable factors making the treatment further challenging. We went ahead with APR followed by post-operative dacarbazine and carboplatin. The patient is currently on the fifth cycle of the drugs. It has been almost two years, and we have not made any significant change in the treatment protocol and the patient is doing well with it, with tolerable side effects. Stoma care is the only concern for the patient, right now, otherwise, he is able to give continuity to his daily routine activities with minimal difficulties.
